# An ISG15-Based High-Throughput Screening Assay for Identification and Characterization of SARS-CoV-2 Inhibitors Targeting Papain-like Protease

**DOI:** 10.3390/v16081239

**Published:** 2024-08-01

**Authors:** Subodh Kumar Samrat, Prashant Kumar, Yuchen Liu, Ke Chen, Hyun Lee, Zhong Li, Yin Chen, Hongmin Li

**Affiliations:** 1 Department of Pharmacology and Toxicology, R Ken Coit College of Pharmacy, The University of Arizona, 1703 E Mabel St, Tucson, AZ 85721, USA; du.pkumar@gmail.com (P.K.); yliu@pharmacy.arizona.edu (Y.L.); kechen2@arizona.edu (K.C.); zli@pharmacy.arizona.edu (Z.L.); ychen@pharmacy.arizona.edu (Y.C.); 2 Department of Pharmaceutical Sciences, College of Pharmacy and Biophysics Core, Research Resources Center, University of Illinois at Chicago, Chicago, IL 60607, USA; danielhl@uic.edu; 3 Department of Chemistry and Biochemistry, College of Science & College of Medicine, The University of Arizona, Tucson, AZ 85721, USA; 4 The BIO5 Institute, The University of Arizona, Tucson, AZ 85721, USA

**Keywords:** SARS-CoV-2, high-throughput screening, PLpro, ISG15, FRET assay

## Abstract

Emergence of newer variants of SARS-CoV-2 underscores the need for effective antivirals to complement the vaccination program in managing COVID-19. The multi-functional papain-like protease (PLpro) of SARS-CoV-2 is an essential viral protein that not only regulates the viral replication but also modulates the host immune system, making it a promising therapeutic target. To this end, we developed an in vitro interferon stimulating gene 15 (ISG15)-based Förster resonance energy transfer (FRET) assay and screened the National Cancer Institute (NCI) Diversity Set VI compound library, which comprises 1584 small molecules. Subsequently, we assessed the PLpro enzymatic activity in the presence of screened molecules. We identified three potential PLpro inhibitors, namely, NSC338106, 651084, and 679525, with IC_50_ values in the range from 3.3 to 6.0 µM. These molecules demonstrated in vitro inhibition of the enzyme activity and exhibited antiviral activity against SARS-CoV-2, with EC_50_ values ranging from 0.4 to 4.6 µM. The molecular docking of all three small molecules to PLpro suggested their specificity towards the enzyme’s active site. Overall, our study contributes promising prospects for further developing potential antivirals to combat SARS-CoV-2 infection.

## 1. Introduction

Emerging variants of SARS-CoV-2 have been causing frequent outbreaks of COVID-19 with varying severity in different parts of the world. While SARS-CoV-2 vaccines have played a crucial role in addressing the pandemic, the resurgence of the disease in vaccinated or previously infected individuals remains a major concern [1,2,3,4]. Reports indicate that individuals infected with the Delta variants exhibit greater disease severity compared to earlier strains like the Alpha and Beta variants [5,6,7]. Therefore, there is an urgent need to develop novel small molecule-based antiviral drugs to combat newly emerging strains of SARS-CoV-2 and reduce disease severity, especially in non-responding and immunocompromised patients.

SARS-CoV-2 belongs to the beta coronavirus genus and possesses a positive-sense, single-stranded RNA genome that encodes four structural proteins, sixteen non-structural proteins (nsps), and ten accessory proteins [8,9,10,11]. The nsps are encoded by two large open reading frames (ORF), ORF1a and ORF1b, which produce two polyproteins, pp1a and pp1ab, upon translation, respectively [12]. The polyprotein pp1a is encoded by ORF1a, while pp1ab is produced through a −1 ribosomal frameshift at a short overlap of the two ORFs [13]. These polyproteins are further proteolytically cleaved by two self-cleaved viral cysteine proteases, nsp3 (PLpro) and nsp5 (3CLpro), resulting in nsp1-nsp11 from pp1a, and nsp10 and nsp12-nsp16 from pp1ab [14]. The nsps, namely, nsp8, nsp9, nsp10, nsp12, nsp13, nsp14, and nsp16 together compose the viral replication–transcription complex (RTC), which plays a vital role in proper genome replication, transcription, and maturation of new virus particles [15,16].

The two viral cysteine proteases are equally indispensable during the viral replication process [17,18]. Cleavage of polyproteins is essential for virus replication. The chymotrypsin-like protease (3CLpro), also known as the main protease (Mpro), cleaves the polyproteins at eleven sites with the recognition sequence “X-(L/F/M)-Q↓(G/A/S)-X” [19], where X refers to any amino acid. Conversely, the papain-like protease (PLpro) cleaves the polyprotein at three sites with the recognition sequence “LXGG↓XX” [20]. PLpro is one of the domains in nsp3 and, besides cleaving the polyproteins, it also performs other essential functions in the viral life cycle [21]. The enzyme is highly conserved and encoded by the genome of all the coronaviruses [22]. While the sequence of PLpro shows very little similarity with human proteases [20], the structural and functional similarity may pose challenges for developing therapeutics effective against multiple coronaviruses [20,23].

PLpro of SARS-CoV-2 effectively modulates the host immune response against virus infection [24]. It also exhibits deubiquitinating activity and cleaves the host Interferon-Stimulating Gene 15 (ISG15) from Interferon Responsive Factor 3 (IRF3), leading to the dysregulation of antiviral inflammatory and interferon responses [21,25]. Research by Ratia et al. has demonstrated that SARS-CoV-2 PLpro displays a marked preference for ISG15 over mono-ubiquitin as a substrate. Their findings reveal that PLpro cleaves ISG15 more efficiently than ubiquitin, based on thorough structural and kinetic studies. These investigations shed light on the distinct ways PLpro interacts with and processes these substrates [26]. The selective action of PLpro on ISG15 suggests that ISG15 can be used as a potent substrate for the development of in vitro assays.

The structures of the PLpros of SARS-CoV and SARS-CoV-2 are well characterized, and structure-based drug discovery has led to the identification of several small molecules, such as GRL0617, which can specifically inhibit the SARS-CoV PLpro [20,27]. GRL-0617 was identified by Ratia et al. through a screening of 50,000 molecules against the PLpro of SARS-CoV [27]. The compound demonstrated an inhibitory concentration (IC_50_) of 0.6 μM against SARS-CoV PLpro and inhibited viral replication in cellular models with an effective concentration (EC_50_) of 14.5 μM [26]. Several other studies have evaluated GRL0617 as an inhibitor of SARS-CoV-2 PLpro, demonstrating its activity against the SARS-CoV-2 PLpro with IC_50_ values in micromolar range [28,29,30,31].

Recently, Tan et al. designed and synthesized 85 noncovalent compounds that inhibited SARS-CoV-2 PLpro, with IC_50_ values ranging from 13.2 to 88.2 nM. Their lead compound, Jun12682, showed efficacy in reducing viral loads and enhancing survival rates in a mouse model infected with SARS-CoV-2, suggesting its potential as an oral antiviral agent [32]. Other reports have also identified small molecule and peptide-based covalent inhibitors targeting SARS-CoV-2 PLpro, such as rac5c, VIR250, and VIR251 [33,34]. However, none of these molecules have been approved for the treatment of COVID-19 patients. Therefore, more efforts are needed, employing novel high-throughput screening (HTS) strategies, to identify clinically effective molecules targeting the viral protease.

Previously, several laboratories reported HTS assays against the SARS-CoV-2 PLpro using peptide-based substrates [28,35,36,37]. However, it is worth noting that peptide substrates may not fully recapitulate the recognition features of the protein substrates of PLpro. In our lab, we have previously performed several HTS campaigns, leading to identification of several small molecule inhibitors against various viral pathogens [38,39,40,41,42,43]. In this manuscript, we report the development of an ISG15-based HTS assay using the fluorescence resonance energy transfer (FRET) technique [44] to identify PLpro inhibitors. We also present the results of a pilot high-throughput screening of the Diversity Set VI compound library, which consists of 1584 small molecule compounds. Through our screening efforts, we successfully identified three molecules with IC_50_ (half-maximum inhibitory concentration) values in the low micromolar range. Additionally, these molecules demonstrated anti-SARS-CoV-2 activity in cell-based assays.

## 2. Materials and Methods

### 2.1. Cell Lines and Viruses

Vero-E6, Vero-CCL81 cell lines used in this study were purchased from American Type Cell Culture (ATCC, Manassas, VA, USA). Vero-E6 cells were cultured in DMEM, with each of the mediums supplemented with 10% FBS and 1% antibiotic mix (penicillin–streptomycin). All cell lines were maintained at 37 °C with 5% CO_2_. The study utilized the severe-acute-respiratory-syndrome-related coronavirus 2 (SARS-CoV-2) strains 2019-n-CoV/USA-WA1/2020 and Omicron (B.1.1.529).

### 2.2. Expression and Purification of SARS-CoV-2 PLpro

Codon-optimized gene insert encoding the SARS-CoV-2 PLpro domain and a C-terminal His-tag was custom synthesized and cloned into the pET28a vector by Gene Universal. His-tagged PLpro protein was expressed and purified as described previously [40]. Briefly, *E. coli* Rosetta (DE3) cells were transformed with the PLpro-expressing plasmid and cultured at 37 °C in LB broth containing 100 ug/mL ampicillin until the absorbance (600 nm) reached 0.6. IPTG was then added at a final concentration of 0.5 mM to the cell culture to induce protein expression for 16 h at 18 °C. The pellet of induced cells was lysed by sonication in a lysis buffer containing 50 mM Tris-HCl (pH 8.5), 150 mM NaCl, and 10 mM imidazole. The lysate supernatant was loaded on Ni-NTA affinity column (Qiagen, Mat. No. 1018244, Gemany). His-tagged PLpro protein was eluted from the column using a lysis buffer containing 300 mM imidazole. Further purification of PLpro protein was achieved by size exclusion chromatography with a Sephadex 75 column. Purified protein was stored at −80 °C in a buffer containing 20 mM Tris-HCl, 100 mM NaCl, and 1 mM TCEP (pH 7.2) at a concentration of 1.8 mg/mL determined using BSA standard (Thermo Fisher scientific, Cat. No. 23209, Waltham, MA).

The catalytic domain of ubiquitin-specific peptidase 25 (USP25) encoding amino acids 157-706 was codon optimized and cloned into a pET28a vector by Gene Universal. A 3C protease-recognition site was designed to replace the thrombin site to facilitate N-terminal His-tag removal. The expression and purification of USP25 was similar to that described above.

### 2.3. Generation of CFP-3C-ISG15-YFP and CFP-Ubiquitin-YFP Substrate

To generate the CFP-3C-ISG15-YFP (CIY) substrate, a codon-optimized gene sequence encoding a fusion protein was synthesized and cloned by Gene Universal. The fusion protein consisted of a Flag-tagged cyan fluorescent protein (CFP), followed by a GAS[KTSAVLQSGFRKME]GGGG-ISG15 linker, where the sequence in brackets represents the 3CLpro recognition sequence, and finally, a yellow fluorescence protein (YFP). This gene sequence was then cloned between the Nde1 and EcoR1 sites of the pET28a vector. The resulting fusion protein contained an N-terminal His-tag. Similarly, CFP-ubiquitin-YFP substrate was also generated by replacing ISG15 with ubiquitin. Purification of the substrates was carried out similarly as described above for PLpro, with the following modifications. After elution from the Ni-NTA column, the purified protein was dialyzed in a buffer containing 20 mM Tris-HCl (pH 8.0), 150 mM NaCl, and 1 mM DTT. Dialyzed CFP-3C-ISG15-YFP proteins were stored in −80 °C at concentration of 1.4 mg/mL determined by using BSA standard. Similarly, a CFP-ubiquitin-YFP substrate was made and stored at concentration of 1.2 mg/mL in −80 °C.

### 2.4. FRET Assay for Determination of Enzymatic Activity of PLpro

The FRET assay was conducted using the SARS-CoV-2 PLpro enzyme and its CFP-3C-ISG15-YFP fusion-protein substrate. A standard curve was established by treating the fusion protein substrate at different concentrations ranging from 0.25 µM to 2.0 µM, with 50 nM of the PLpro enzyme. These mixtures were incubated for 30 min at room temperature, and the fluorescence intensities were measured at 530 nm (*I*_530_) and 475 nm (*I*_475_), respectively. The FRET ratio was calculated as *I*_530_/*I*_475_. Progress of the reaction was continuously monitored until all the substrates were consumed, and the reaction reached a plateau. Higher values of the FRET ratio suggest a greater FRET efficiency, while ratio below 2.0 suggests the lack of FRET. A graph of the FRET ratio versus substrate concentration in log scale was plotted using the PRISM 8.0 software (GraphPad, La Jolla, CA, USA). A comparative analysis was also performed for a commercially available Z-RLRGG-AMC peptide substrate (Bachem, Cat. No. 4027158, Torrance, CA, USA) and our CIY based protein substrate using the GRL0617 inhibitor as a control.

### 2.5. Determination of Inhibitory Activity of the Selected Compounds on PLpro and USP25

Inhibitor screening against the SARS-CoV-2 PLpro was conducted using a FRET-based assay with the CFP-3C-ISG15-YFP substrate. The screening assay was performed in a reaction buffer composed of 20 mM Tris (pH 7.4), 150 mM NaCl, 1 mM EDTA, 1 mM DTT, 0.4 µM CFP-3C-ISG15-YFP substrate, and 40 nM PLpro. The HTS against the NCI Diversity Set VI library containing 1584 compounds spread across 20 plates was carried out in 96-well black polypropylene plates with a reaction volume of 100 µL per well. Prior to initiating the reaction, PLpro was incubated with either DMSO (control) or the inhibitor at 30 µM for 30 min at ambient temperature. Subsequently, the CFP-3C-ISG15-YFP substrate was added to the reaction mixture. After 35 min of incubation, the 530/475 nm fluorescence ratio was calculated to analyze the inhibitory effect of compounds on the enzymatic activity of PLpro.

The Z-factor, signal to background ratio, and coefficient variance were calculated using the standard deviations and the FRET values of samples in the absence and presence of positive control inhibitor GRL0617 (25 µM), as previously described [38,39,41]. To determine the inhibitory activity (IC_50_) of the selected compounds against PLpro, the FRET assay, as described above, was conducted using a concentration series of the inhibitors ranging from 120 µM to 0.4 μM. To eliminate the promiscuous compound, we additionally ascertained the inhibitory activity IC_50_ of selected compounds against PLpro. The FRET assay, outlined previously, was employed, employing a range of inhibitor concentrations in a reaction buffer consisting of 20 mM HEPES (pH 7.5), 5 mM DTT, and 0.01% Tween-20.

To assess selectivity, we conducted a selectivity assay on selected compounds against a representative human deubiquitinase (DUB), USP25, using the CFP-ubiquitin-YFP substrate, as described above. The experimental condition was similar to that for the PLpro assay, with the exception that USP25 and the CFP-ubiquitin-YFP substrate were employed instead of PLpro and the CIY substrate.

### 2.6. Inhibition Mechanism of SARS-CoV-2 PLpro Inhibitors

Further, to investigate how the identified molecules inhibit PLpro in vitro, reaction mixtures of 50 μL containing 40 nM of PLpro and varying concentrations of small molecules were combined with different concentrations of peptide-AMC substrate. The initial reaction rates were determined using the FRET assay method. The inhibition mechanism of small molecules on PLpro was analyzed using the Lineweaver–Burk plot.

### 2.7. Cellular Cytotoxicity

The cytotoxicity of selected inhibitors was assessed on Vero CCL81 cells using the Cell Counting Kit (CCK-8) from GLPBIO. Cells were seeded in a 96-well plate at a density of 2 × 10^5^ cells per well in 100 µL DMEM medium containing 10% FBS. The cells were then cultured at 37 °C in a 5% CO_2_ atmosphere until they reached approximately 90% confluency. Next, the cells were treated with different concentrations of inhibitors for 48 h. Then, 10 µL of CCK-8 reagent was added to each well, followed by an incubation of up to 4 h. The absorbance at 460 nm was measured using a BioTek Synergy H1 microplate reader. CC_50_ (50% cytotoxic concentration) values were calculated by plotting the dose–response curve using the PRISM 8 software.

### 2.8. Antiviral Plaque Reduction Assay (PRA)

The antiviral activity of the selected PLpro inhibitors was determined on Vero-E6 cells. The cells were seeded at a cell density of 15,000 cells per well in a 96-well flat-bottom tissue culture plate and cultured in DMEM medium containing 10% FBS at 37 °C/5% CO_2_ for 24 h. Once the cells reached 80–90% confluency, they were washed twice with 100 µL of sterile 1X PBS. Subsequently, the cells were separately infected for 1 h with 0.01 MOI of the wild-type WA1 strain of SARS-CoV-2 in the presence of each of the three selected compounds at concentrations ranging from 0.3 µM to 25 µM of. At 1 h post-infection, the cells were covered with 100 µL of 1% methylcellulose overlay and further incubated for 72 h at 37 °C/5% CO_2_. Then, the cells were fixed with 10% neutral buffered formalin for 30 min. Fixed cells were thoroughly washed with water and stained with 0.2% crystal violet for 10 min. The resulting plaques were counted. EC_50_ (half-maximum effective concentration) values were calculated using the GraphPad Prism 9.0. All the experiments involving live SARS-CoV-2 virus were conducted in a BSL-3 facility at University of Arizona to ensure proper containment and safety measures.

### 2.9. Antiviral Immunofluorescence Assay (IFA)

The Vero-E6 cells were grown to a confluency of ≈70% in DMEM medium containing 10% FBS (D10). The selected inhibitor compounds were diluted (1:2) in D10 medium to create a concentration range from 100 µM to 12 µM. A total of 60 µL of each compound at each concentration was dispensed into designated wells of 96-well round-bottom plates. The SARS-CoV-2 strains, WA1 and Omicron, were diluted in D10 medium to achieve a MOI of 0.01 and 0.02, respectively. Subsequently, 60 µL of the diluted virus was added to each well containing the compounds, resulting in a final volume of 120 µL in each well. Then, 100 µL of the compound–virus mixture was added to the corresponding wells of a 96-well tissue culture plate containing Vero-E6 cells and incubated at 37 °C/5% CO_2_ for up to 48 h. The cells were then fixed with 4% normal buffered formalin for 30 min and thoroughly washed with running water. Immunofluorescence assay (IFA) was performed following the standard protocol. Briefly, the fixed cells were washed thrice with 1X PBS and permeabilized with 0.1% Triton X 100 (in PBS) for 10 min at room temperature. The cells were washed again and blocked with 2% BSA for 1 h at room temperature. Subsequently, the cells were incubated overnight at 4 °C with a primary antibody (mouse anti-NP antibody) diluted at 1:100, followed by three washes with 1X PBS. Next, the cells were incubated at room temperature for 1 h with a secondary antibody (anti-mouse Alexa Fluo488) diluted at 1:2000, and then they were stained with DAPI at 1:2000 dilution. The cells were imaged using the BioTek Cytation 5 Cell Imaging Multi-Mode Reader.

### 2.10. Inhibition of Cleavage of Protein Substrate by Inhibitors

To visualize the inhibitory effect of each inhibitor on PLpro, 1 μM of the PLpro enzyme was separately incubated with 60 µM, 30 µM, and 15 µM of each inhibitor in a Tris buffer (20 mM Tris (pH 7.4), 150 mM NaCl, 1 mM DTT, and 1 mM EDTA) for 30 min. Then, the CFP-3C-ISG15-YFP protein substrate was added to the reaction mixture at a final concentration of 1.5 µM, and the reaction mixture was further incubated for 30 min, followed by SDS-PAGE analysis.

### 2.11. Surface Plasmon Resonance

The SARS-CoV-2 PLpro (pp1ab amino acids 1564-1878) was purified as described in this section. The purified PLpro enzyme was initially stored in buffer containing 50 mM Tris-HCl (pH 8.5), 150 mM NaCl, 10 mM DTT, 5% glycerol, and buffer exchanged with HBSP buffer (10 mM HEPES (pH 7.4), 150 mM NaCl, 0.05% Tween-20) prior to immobilization. Standard amine-coupling was used to immobilize the PLpro on a CM5 sensor chip with running buffer HBSP using a Biacore 8K instrument. Unmodified channels were used as reference control surfaces. The PLpro enzyme was diluted in 10 mM sodium acetate (pH 5.5) and immobilized after sensor surface activation with 1-ethyl-3-(3-dimethylaminopropyl) carbodiimide hydrochloride (EDC)/N-hydroxy succinimide (NHS) mixture followed by ethanolamine (pH 8.5) blocking on unoccupied surface area. Testing compounds were initially prepared as 10 mM DMSO stock solutions, and compound solutions with a series of increasing concentrations (0.046–100 µM at 3-fold dilution) were applied at a 30 µL/min flow rate at 25 °C. The multi-cycle kinetic method was run, and real-time response units were monitored. Sensorgrams were double referenced with the reference channel and zero concentration responses and fitted with 1 to a 1 Langmuir kinetic model embedded in the Biacore Insight software v4.0.8, producing two rate constants, *k*_a_ and *k*_d_. Equilibrium dissociation constant (*K_D_*) values, often referring to as binding affinity, were calculated from two determined rate constants (*K_D_* = *k*_d_/*k*_a_). The steady-state affinity model was also used to fit the data at equilibrium to determine *K_D_*.

### 2.12. Molecular Docking

The potential inhibitors NSC338106, 651084, and 679525 were docked within the crystal structure of PLpro of SARS-CoV-2, obtained from the Protein Data Bank (PDB ID:7JRN), using Schrodinger Maestro Version 13.3, released in March 2022, running on a Windows-x64 platform.

The crystal structure 7JRN was imported to Schrodinger Maestro. Protein pre-processing was carried out using the Protein Preparation Workflow. Protein pre-processing settings included cap termini, assigning bond orders using the CCD Database, replacing hydrogens, filling in missing loops (using PRIME), generating het states using EPIK pH 7.4 +/− 2.0, and filling missing side chains. To eliminate overlapping hydrogens, we optimized H-bond assignments. The optimization parameters covered H-bond assignments, sampling water orientations, and using PROPKA (pH 7.4) to predict pKa values of ionizable groups, which is crucial because the binding affinity (i.e., the binding free energy) depends on the protonation states of the ionizable residues and functional groups in the ligand binding site. Water cleanup and restrained minimization were conducted to converge heavy atoms (non-hydrogen) to an RMSD 0.30 Å. We applied the OPLS4 Force Field, removed waters within 5 Å of ligands, and eliminated waters with fewer than 3 bonds to non-waters.

The bound inhibitor GRL0617 in 7JRN was removed prior to binding pocket evaluation using the Protein Preparation Workflow in Schrodinger. Binding site evaluation was performed using SiteMap to identify top-ranked potential ligand binding sites. Each binding site required at least 15 site points. The top five binding sites were reported, and the site map at 4 Å from nearest site point was cropped for characterization (Figure S5). The putative binding sites with a D-score higher than 0.8 were considered as a druggable binding pocket. Three out of five pockets satisfied this criterion. The three pockets, termed the active site and allosteric sites 1 and 2, were centered around the PLpro residues TYR273, ASP76, and TYR83, respectively (Figure S6).

Ligands NSC338106, 651084, and 679525 were prepared using LigPrep to generate ionization states at pH 7.4 (+/− 2.0), including processes such as EPIK, desalting, tautomer generation, and retaining specified chiralities. Solvents were removed from the structure for docking and analysis.

Docking was performed using the Induced Fit Docking protocol with the prepared ligands. Three ligand-binding boxes for the three putative ligand binding sites (active site, allosteric sites 1 and 2) were created around the centroid of PLpro residues TYR273, ASP76, and TYR83 to accommodate ligands within a 30 Å, 20 Å, and 20 Å radius, respectively (Figure S6). These grid boxes provided space large enough to include the whole putative binding pockets (Figure S6). Ligand preparation included sampling ring conformations for all defined functional groups, with an energy window of 2.5 Kcal/mol. Glide docking was conducted with receptor van der Waals scaling set to 0.5, ligand van der Waals scaling also set to 0.5, and maximum number of 20 possible poses per ligand. Residues within 5 Å of ligand poses were refined, and side chains were optimized. Ligands were redocked into structures with energies within 30 kcal/mol of the best structure, and those within the top 10 structures overall in standard precision mode were considered. Molecular docking results were ranked based on glide docking scores, with the best structure selected for subsequent structural analysis.

## 3. Results

### 3.1. ISG15-Based PLpro Assay

PLpro serves a dual role, functioning as a viral protease for processing the viral polyprotein and as a promoter of viral replication by interfering with the host’s innate immune response [45,46]. It achieves this interference by breaking the C-terminal RLRGG isopeptide bond that attaches ISG15 to lysine side chains of host proteins, acting as a deubiquitinase (DUB) toward ISG15-modified proteins [15,29,47]. ISG15 is a critical component of the host antiviral response and is stimulated by IFNα [48].

**Figure 1:**
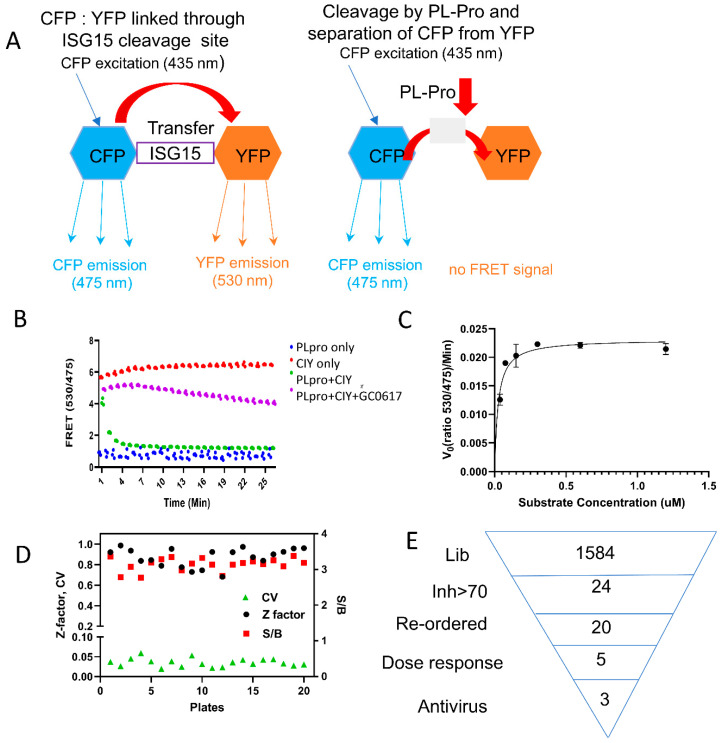
Measurements of PLpro activity and HTS using the ISG15-based assay. (**A**) The ISG15 FRET assay relies on FRET from CFP to YFP. In our construct, CFP and YFP, the FRET pairs, are fused together with the ISG15 protein, which is sensitive to PLpro cleavage. Consequently, the natural FRET signal can be disrupted when the PLpro enzyme cleaves and separates CFP-ISG15 from YFP. (**B**) The ratio of fluorescence at 530/475 is shown for different conditions: PLpro alone (blue), CFP-3C-ISG15-YFP (CIY) substrate alone (red), CIY substrate with PLpro (green), and CIY substrate with PLpro and GRL0617 (purple). (**C**) The kinetic analysis of PLpro and the CIY substrate. n = 3. (**D**) The Z’ score, S/B ratio, and CV for HTS. (**E**) The screening flowchart.

To identify PLpro inhibitors, we developed an ISG15-based PLpro assay. In this assay, CFP and YFP were linked through ISG15, which is a natural protein substrate of PLpro [21], creating a FRET substrate to measure PLpro enzyme activity (Figure 1A). CFP and YFP are a commonly used pair of proteins in FRET experiments, due to their well-separated excitation and emission spectra, enabling efficient energy transfer between donor and acceptor [49]. Upon excitation of CFP at 435 nm, emissions can be detected at 475 nm (representing the CFP emission peak), and in the presence of the FRET pair YFP, emissions can also be observed at 530 nm (indicating the YFP emission peak) [49,50]. Cleavage of the ISG15 linker between CFP and YFP by PLpro disrupts the FRET between donor (CFP) and acceptor (YFP). FRET efficiency is quantified by measuring the ratio of fluorescence intensity at 530 nm (*I_530_*) to that at 475 nm (*I_475_*).

We purified both the PLpro enzyme and the CFP-3C-ISG15-YFP (CIY)-based protein substrate. FRET efficiency, defined as the ratio (*I*_530_/*I*_475_) of fluorescence intensities at 530 nm to 475 nm, was then used to analyze the inhibitory effect of compounds on the enzymatic activity of PLpro. Higher values of the FRET ratio indicate greater FRET efficiency, while ratios below 2.0 suggest the absence of FRET. Our kinetic results indicated that significant FRET reductions were observed for CFP-3C-ISG15-YFP in the presence of PLpro over time, suggesting that PLpro digests CFP-3C-ISG15-YFP in a time-dependent manner (Figure 1B). We determined the initial velocity of the cleavage based on the change of the 530/475 nm ratio and then plotted it against the substrate concentrations, as previously described [51]. The data exhibited a clear dose–response relationship with the substrate. From the fitted curve, the key kinetic parameters *K_m_* were calculated to be 0.02 µM (Figure 1C). Furthermore, our results demonstrate that the signal-to-background (S/B) ratio is greater than 3.

To validate the assay, a known PLpro inhibitor, GRL0617, was used as a positive control [27,37]. We further compared our CIY-based protein substrate with a previously known peptide-AMC substrate using GRL0617 as a control inhibitor. Our results suggested that the compound inhibited the PLpro activity with IC_50_ values of 1.6 µM and 1.9 µM for the peptide-based AMC substrate and our protein-based substrate, respectively, thus exhibiting similar sensitivity (Figure S1).

### 3.2. High-Throughput Screening

The NCI Diversity Set VI library, comprising 1584 compounds distributed across twenty 96-well plates, was screened to identify PLpro inhibitors, using the ISG15-based FRET assay as described above. Each compound was tested at a fixed concentration of 30 µM during the screening. DMSO served as a negative control for each plate screening, while GRL0617 at 30 µM was employed as a positive control for inhibition. The quality of the HTS assay was assessed by analyzing the Z’-factor, S/B ratio, and coefficient of variation (CV) for each plate [52]. The average values obtained were 0.87, 3.1, and 3.5%, respectively (Figure 1D,E and Figure S2), confirming the high quality of the HTS assay.

**Figure 2:**
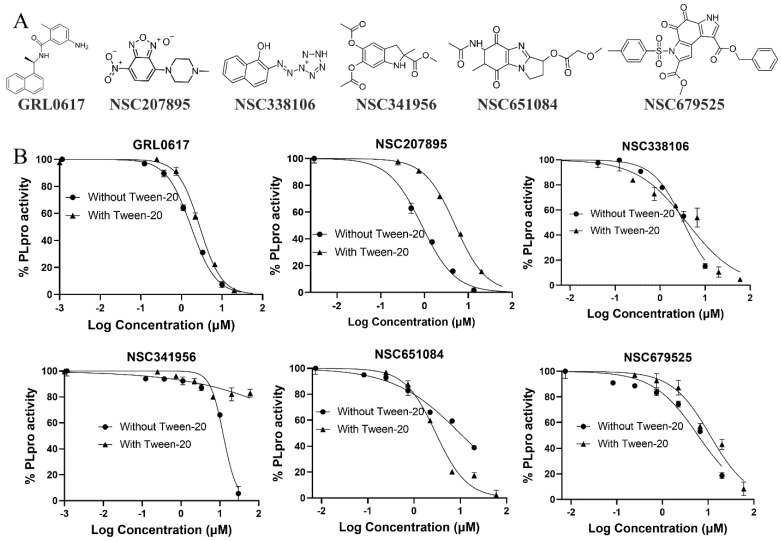
Identified candidate PLpro inhibitors. (**A**) Structures of GRL0617 and the identified small molecule inhibitors: NSC207895, 338106, 341956, 651084, and 679525. (**B**) Dose–response curves for small molecule inhibitors tested against PLpro using the ISG15-based assay. The ISG15−based CIY substrate was employed, and its cleavage by PLpro was assessed in the presence of small molecule inhibitors in the presence or absence of 0.01% Tween−20. GRL0617 served as the positive control inhibitor, and the IC_50_ of each inhibitor was determined. All experiments were performed in triplicate, and all data are expressed as the mean ± standard deviation.

During the screening process, twenty-four compounds were identified, of which exhibited over 50% inhibition of the PLpro activity. Subsequent testing confirmed that five compounds, specifically NSC207895, 338106, 341956, 679525, and 651084, demonstrated dose-dependent inhibition of PLpro activity (Figure 2A). The IC_50_ values for these five compounds ranged from 0.9 µM to 12.4 µM (Figure 2B, Table 1). These results suggest that these compounds exhibit promising inhibitory potential as PLpro inhibitors and warrant further exploration as potential candidates for the development of antiviral treatments against SARS-CoV-2.

We next conducted inhibition analysis in the presence of 0.01% Tween-20 to triage promiscuous compounds, using GRL0617 as a positive control. Our results revealed that detergent did not dramatically affect the inhibition activity of compounds NSC338106, NSC651084, and NSC679525, nor the control inhibitor GRL0617. In contrast, compounds NSC207895 and NSC341956 were significantly affected by Tween-20, with IC_50_ values increasing more than 5- and 16-fold, respectively. The findings suggest that compounds NSC207895 and NSC341956 might be promiscuous inhibitors, whereas NSC338106, NSC651084, and NSC679525 were true hits and were further investigated for antiviral efficacy (Table 1).

We also conducted a time inhibition trial to determine if inhibition is due to enzyme modification. However, we observed that the IC_50_ did not significantly change with either a 5 min or 60 min preincubation with the enzyme (Figure S3, Table S1).

### 3.3. Specificity

Since PLpro substrates are also substrates of human deubiquitinase (DUBs), it is necessary to assess if the hit compounds inhibit human DUB activity. We expressed and purified the DUB domain of a representative DUB, USP25, and generated a USP25 CFP-ubiquitin-YFP (CUY) substrate similar to CIY. Using the CUY substrate, we conducted a dose-dependent inhibition analysis of USP25 activity by our hit compounds. Our results showed that NSC338106, NSC651084, and NSC679525 inhibited USP25 activity with IC_50-USP25_ values of 37 µM, 23 µM, and 41 µM, respectively (Figure S4, Table 1), resulting in a selectivity index (SI), defined as IC_50-USP25_/IC_50-PLpro_, ranging from 4.7 to 11.2. The selectivity index indicates that NSC338106, NSC651084, and NSC679525 are relatively selective for PLpro over USP25, with selectivity indices >4.7. This suggests that while these compounds show promising inhibition of PLpro, their specificity could be further improved to reduce potential off-target effects on human DUBs.

### 3.4. Inhibition of SARS-CoV-2 Replication by PLpro Inhibitors

**Figure 3:**
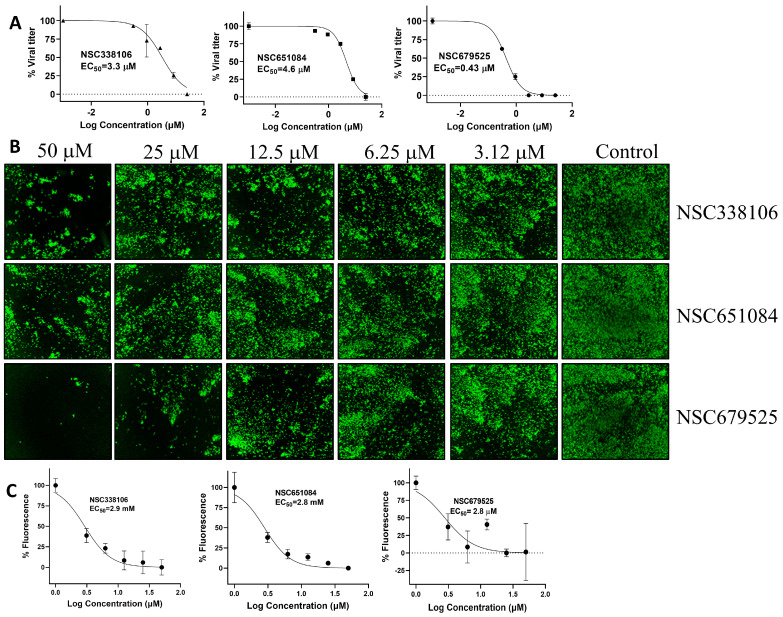
Antiviral activities. (**A**) Curve fitting of data from the plaque reduction assay of small molecule PLpro inhibitors against SARS-CoV-2. Dose–response titrations were carried out in 96-well plates with Vero-E6 cells (1.5 × 10^4^ cells/well). On the following day, the cells were infected with SARS-CoV-2 (MOI: 0.01) while exposed to increasing concentrations of the compounds (ranging from 25 μM to 0.3 μM). Virus infection was quantified using a plaque reduction assay after 24 h. The signals from the samples were normalized using signals from the DMSO control wells. The data represent the mean ± standard error from triplicate experiments, N = 3. (**B**) Representative images of immunofluorescence assay (IFA) for dose-dependent inhibition of the Washington Strain (WA1) of SARS-CoV-2 treated with decreasing concentrations (50 µM to 3.1 µM) of NSC338106, 651084, and 679525. Vero−E6 cells were infected with the virus, treated with compounds at indicated concentrations for 24 h, fixed, and immunolabeled with a primary SARS-CoV-2 nucleocapsid monoclonal antibody and a goat anti−mouse secondary Alexa−488 antibody. (**C**) Dose−dependent inhibition of protein expression of the WA1 of SARS-CoV-2 treated with decreasing concentrations (50 µM to 3.1 µM) of compounds. Normalized data obtained from IFA as shown in panel (**B**) was plotted against concentration of compounds. The intensities of Alexa−488 positive cells for the DMSO control were set as 100%, N = 6.

The antiviral activity of the inhibitors was conducted using a plaque reduction assay (PRA) in Vero-E6 cells. The cells infected the WA1 strain of SARS-CoV-2 at 0.01 MOI while exposed to decreasing concentrations of each of the candidate compounds, ranging from 25 µM to 0.3 µM. After 72 h of post-infection (p.i.), plaques were counted to determine the viral titer and EC_50_ value of each compound. Among the compounds tested, NSC679525 exhibited the highest efficacy in inhibiting viral replication with an EC_50_ of 0.4 µM. In comparison, NSC338106 and 651084 showed relatively higher EC_50_ values of 3.3 µM and 4.6 µM, respectively (Figure 3A). These results indicate that NSC679525 displays potent antiviral activity against SARS-CoV-2, with a significantly lower EC_50_ value, making it a promising candidate for further evaluation and potential development as an antiviral agent against the virus.

We further investigated the efficacy of the PLpro inhibitors in inhibiting the replication of SARS-CoV-2 through an immunofluorescence assay (IFA) conducted on virus-infected Vero-E6 cells in a 96-well plate. Vero-E6 cells were infected separately the wild-type (WT) WA1 strain at 0.01 MOI and the Omicron strain at 0.02 MOI, in the presence of concentrations ranging from 50 µM to 1 µM of the three candidate compounds. At 24 h p.i. for the WA1 strain and 72 h p.i. for the Omicron strain, the infected Vero-E6 cells were fixed. The impact of the compounds on viral protein expression was assessed using the IFA method. The proportion of infected cells in each well was observed using a primary antibody against viral nucleoprotein [SARS-CoV-2 anti-N (E16C), Cat. No. PIMA17403, Thermo Fisher scientific, Waltham, MA, USA] and a secondary fluorescent antibody [goat anti-mouse IgG (H&L, DyLight 488 Conjugate, Part No. gtxMu-003-E488NHSX, ImmunoReagent, Inc, Raleigh, NC, USA). The results revealed that the compounds exhibited concentration-dependent inhibition of virus infection for the WA1 strain (Figure 3B). The EC_50_ values of the compounds were determined based on the count of infected cells. Specifically, compounds NSC338106, 651084, and 679525 exhibited comparable EC_50_ values of 2.9 µM, 2.8 µM, and 2.8 µM, respectively (Figure 3C).

**Figure 4:**
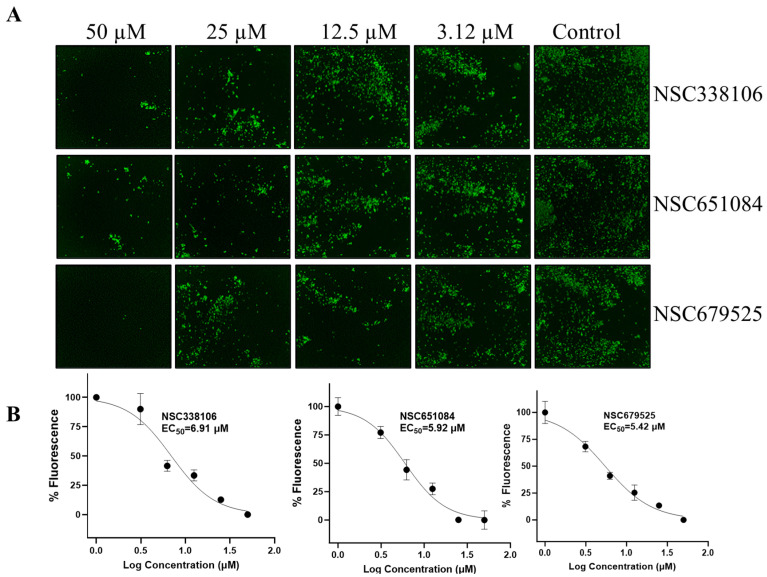
Inhibition of the Omicron strain of SARS-CoV-2. (**A**) Immunofluorescence assay (IFA) for dose-dependent inhibition of the Omicron strain of SARS-CoV-2 treated with decreasing concentrations (50 µM to 1.6 µM) of compounds. Vero-E6 cells were infected with the virus, treated with compounds at indicated concentrations for 48 h, fixed, and immunolabeled with a primary SARS-CoV-2 nucleocapsid monoclonal antibody and a goat anti-mouse secondary Alexa-488 antibody. (**B**) Quantification of the IFA data shown in panel (**A**). Normalized data obtained from IFA was plotted against concentration of compounds. The intensities of Alexa-488-positive cells for the DMSO control were set as 100%, N = 3.

We further evaluated the inhibition of the Omicron strain of SARS-CoV-2 by these compounds (Figure 4). The data demonstrate dose-dependent inhibition of the Omicron stain by NSC338106, 651084, and 679525. These findings underscore the potential of these compounds as effective inhibitors against the WA1 and Omicron variants of the SARS-CoV-2 virus. The observed dose–response relationship suggests that increasing concentrations of NSC679525 resulted in progressively stronger inhibition of viral replication for the Omicron strain, highlighting its promising therapeutic potential in combating this highly transmissible variant.

### 3.5. Cytotoxicity of Candidate PLpro Inhibitors

**Figure 5:**
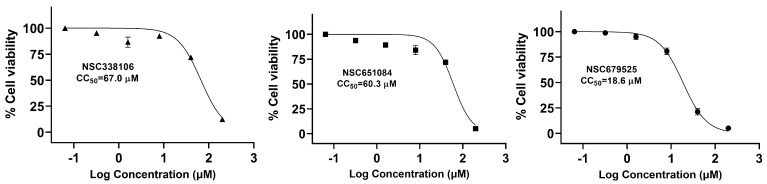
Cytotoxicity activity of NSC338106, 651084, and 679525. Vero−E6 cells were incubated with various concentrations of compounds, and then viability was assayed at 48 h of incubation, using the WST assay, N = 3.

We assessed the cytotoxicity of these three compounds in Vero-CCL81 cells. The cells were treated with varying concentrations of the compounds, and cell viability was determined by the WST assay, which utilizes 2-(2-methoxy-4-nitrophenyl)-3-(4-nitrophenyl)-5-(2,4-disulfophenyl)-2H-tetrazolium, monosodium salt. The CC_50_ values of NSC338106, 651084, and 679525 were determined to be 67.0 µM, 60.3 µM, and 18.6 µM, respectively (Figure 5). These values substantially exceeded their respective EC_50_ values (Figure 3, Table 1). This suggests that the tested compounds possess acceptable cytotoxicity profiles, with CC_50_ values significantly higher than their EC_50_ values. Consequently, it is reasonable to conclude that the compounds’ inhibitory effects on SARS-CoV-2 are not primarily attributable to non-specific cytotoxicity, positioning them as promising candidates for further evaluation as potential antiviral agents.

### 3.6. PAGE Analysis for Cleavage of the CFP-3C-ISG15-YFP Substrate by PLpro

To confirm the mechanism of action, we conducted an analysis using a PAGE gel to visualize the cleavage of the CIY substrate by PLpro and the inhibition of this cleavage by the candidate compounds. In this analysis, we used the CIY substrate at a concentration of 1.5 µM, which was digested with 1.0 µM of PLpro in presence of various concentrations of the inhibitors. GRL0617 was used as the positive control inhibitor to assess the enzymatic activity of PLpro. The digestion of the substrate was analyzed by SDS-PAGE, where the uncleaved CIY substrate appeared as a band at 70 kDa, and the cleaved product emerged at 47 kDa.

**Figure 6:**
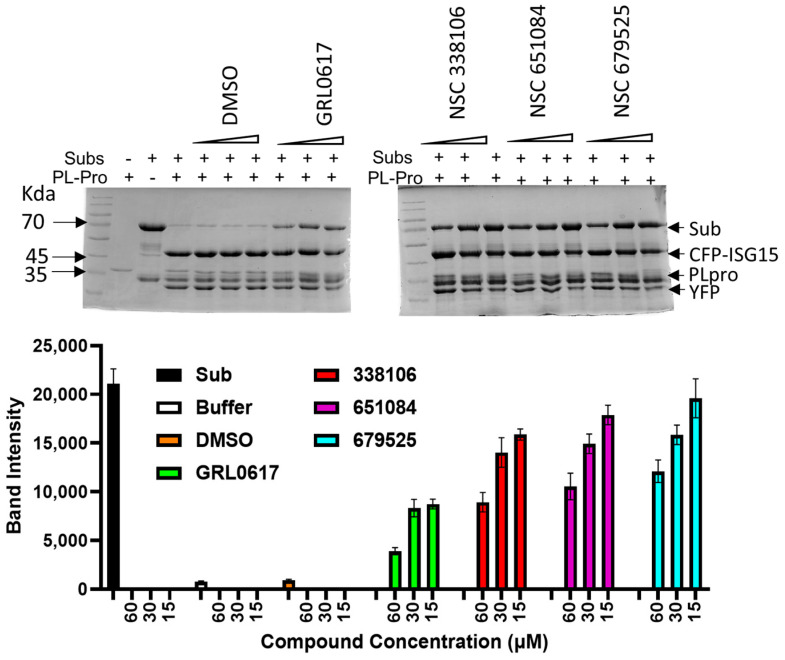
Inhibition of the CIY substrate cleavage by the SARS-CoV-2 PLpro inhibitors. A total of 1 µM of the PLpro protein was incubated for 30 min with increasing concentrations of each inhibitor separately in a Tris buffer. After a 30 min incubation, the CIY protein substrate was added and further incubated for one hour, followed by SDS−PAGE analysis. GRL0617 was used as the positive control. The uncleaved substrate corresponds to 70 kDa, and the cleaved product is 47 kDa. The remaining bands are impurities of protein. All inhibitors were used at 60 µM, 30 µM, and 15 µM concentrations. Gel quantification is shown in the lower panel, N = 4.

As shown in Figure 6, the enzymatic activity of PLpro decreased in proportion to the increasing concentrations of each of the three inhibitors. The observation suggests that the inhibitors effectively impeded the cleavage of the CIY substrate by PLpro, leading to a reduction in the formation of the cleaved product. The dose–response relationship illustrated in the figure underscores the inhibitory effects of the tested compounds, emphasizing their potential as promising inhibitors of PLpro enzymatic activity.

### 3.7. Analysis of Bimolecular Interactions between PLpro and Inhibitors

**Figure 7:**
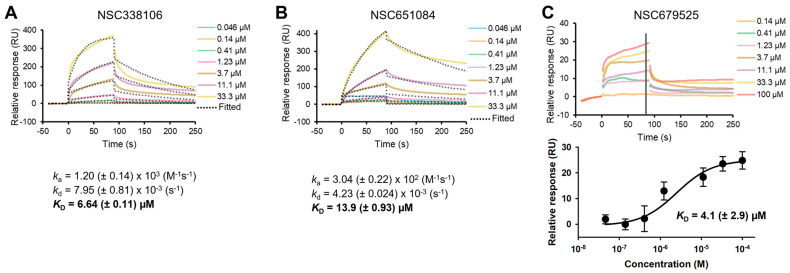
Surface plasmon resonance (SPR) for binding affinity of compounds to recombinant SARS-CoV-2 PLpro. Equilibrium dissociation constant (*K_D_*) values were determined using both the 1:1 Langmuir kinetic model and the steady−state affinity model, which measure the respective association (*K_a_*) and dissociation (*K_d_*) rates of each compound to fit the data from titrating 100−0.1 μM for NSC338106 (**A**), NSC651084 (**B**), and NSC679525 (**C**) against immobilized PLpro.

We conducted an in vitro binding analysis to investigate the direct interaction of these inhibitors with our target PLpro enzyme. Purified PLpro enzyme was immobilized on a CM5 sensor surface, and each inhibitor was applied at a series of increasing concentrations. Our results demonstrated that all three inhibitors indeed exhibited binding with varying equilibrium dissociation constant (*K_D_*), often referred to as binding affinity, ranging from 4.1 µM to 13.9 µM (Figure 7). Among these, two inhibitors, NSC338106 and NSC651084, exhibited a slow association rate (*k*_a_) and slow dissociation rate (*k*_d_), resulting in *K_D_* values at equilibrium of 6.6 µM and 13.9 µM, respectively (Figure 7A,B). In contrast, NSC679525 displayed much faster *k*_a_ and *k*_d_, yielding *K_D_* values of 4.1 µM (Figure 7C). The binding affinities of three inhibitors were comparable with the IC_50_ values within a 2–3-fold difference range (Figure 7C).

### 3.8. Mechanism of Inhibition

**Figure 8:**
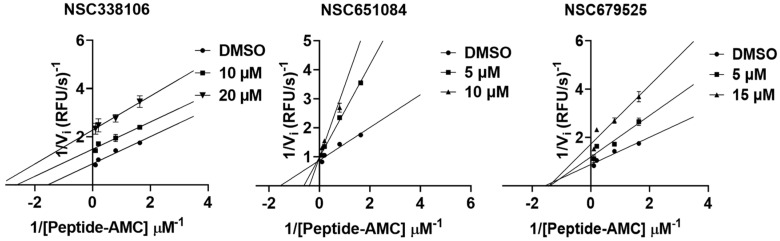
Mechanism for inhibition of SARS-CoV-2 PLpro. Lineweaver–Burk plots for inhibition of the SARS-CoV-2 PLpro by NSC651084, NSC338106, and NSC679525. The SARS-CoV-2 PLpro at 40 nM was mixed with DMSO, NSC651084, NSC338106, and NSC679525 with varying concentrations. Peptide-based AMC substrate was added at various concentrations (1 μM−60 μM).

We next conducted enzyme kinetic inhibition analysis to investigate the mechanism of inhibition. The Lineweaver–Burk plot analysis resulted in a set of straight lines, from which we determined the inhibitory mechanisms of the three compounds (Figure 8). Our results suggest that compounds NSC679525 and NSC651084 showed non-completive and competitive inhibition, respectively, while compound NSC338106 exhibited uncompetitive inhibition.

### 3.9. Molecular Modelling

**Figure 9:**
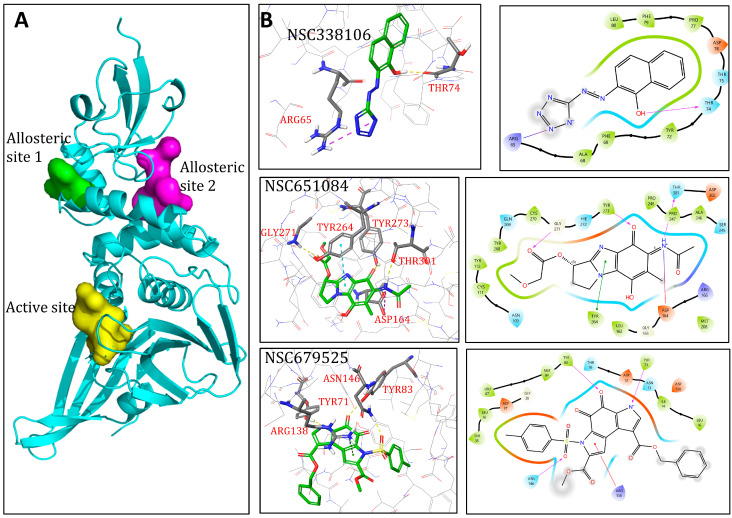
Binding models of candidate inhibitors to the SARS-CoV-2 PLpro in the putative ligand binding sites. (**A**) Overall locations of the three binding sites are as follows: the active site is colored yellow, allosteric site 1 is colored green, and allosteric site 2 is colored magenta. (**B**) Detailed molecular interactions between inhibitors and predicted binding site of PLpro. NSC338106 is docked into allosteric site 1, NSC651084 is docked into the active site, and NSC679525 is docked into allosteric site 2. The right panels show 2D interaction maps where π–π interactions are indicated in green, π–cation interactions in red, salt bridges in gradient blue-red, and hydrogen bonding in purple, with arrows indicating the role as donor or acceptor. The left panels display 3D interactions, with dashed lines indicating different interaction types by color: yellow for hydrogen bonding, purple for salt bridges, green for π–cation interactions, and cyan for π–π interactions.

To further investigate the mechanism of inhibition and investigate the binding modality and possible mechanism of enzyme inhibition, we conducted in silico docking of all three candidate compounds using Schrodinger Maestro. These studies were based on the crystal structure of PLpro of SARS-CoV-2 (PDB ID:7JRN). Initially, the inhibitor GRL0617 was removed from crystal structure. Then, Maestro SiteMap was employed to identify and characterize putative druggable binding sites. Although SiteMap calculated five potential binding pockets, only three, namely, active site and allosteric sites 1 and 2 (Figure 9A,B and Figure S5A–C, Table S2), had D-scores higher than 0.8, a threshold to indicate druggable binding pockets. Subsequently, three binding sites were thoroughly characterized. Upon aligning these putative binding sites with the PLpro crystal structure, it was observed that the active site exhibited the highest scores in both Site and Druggability metrics (Table S2).

The active site comprises residues K157, E161, L162, G163, D164, V165, R166, E167, M208, A246, P247, P248, Y264, Y268, Q269, Y273, T301, and D302, with a volume of 171.5 Å^2^ (Figures S5A,D and S6A). Residue Y273 serves as the center of the active site, around which a grid box with a 30 Å radius is created for docking. Additionally, two allosteric sites were delineated, with allosteric site 2 notably positioned at the dimerization interface (Figure S5C). Allosteric site 1 is composed of residues L58, P59, R65, V66, A68, F69, Y72, T74, T75, D76, P77, F79, and L80, with a volume of 87.8 Å^2^ (Figures S5B,E and S6B). Residue D76 serves as the center of allosteric site 1, around which a grid box with a 20 Å radius is created for docking. Allosteric site 2 is composed of residues D12, N13, Y35, L36, D37, K53, T54, F55, Y56, Y71, Y72, Y83, L87, K91, A131, D134, A135, R138, E143, A145, N146, and L150, with a volume of 182.8 Å^2^ (Figures S5C,F and S6C). Residue Y83 is used as the center of allosteric site 2, around which a grid box with a 20 Å radius is created for docking (Figures S5F and S6). Following this, all three compounds underwent docking within these putative binding pockets using an induced-fit model. The model yielding the most favorable glide docking score was selected for further mechanistic investigation (Table 2). Based on the results, the following interactions were observed (Figure 9).

Based on the docking scores (Table 2), NSC338106 prefers docking into allosteric site 1 (Figure 9B, top panel). NSC338106 exhibits a tetrazole group that engages in a cation–π interaction with R65. The hydroxyl group in the naphthalene group forms a hydrogen bond with T74 of PLpro. NSC338106 also shows potential for docking into allosteric site 2 (Figure S7), with a docking score slightly higher than that for allosteric site 1. The tetrazole group forms a π–π interaction with TYR56. The hydroxyl group in the naphthalene moiety forms two hydrogen bonds, acting as a donor with TYR56 and as an acceptor with TYR83.

NSC651084 prefers docking into the active site (Figure 9B, middle panel). NSC651084 features an acetamide group that forms a hydrogen bond with T301 and a salt bridge with D164. The oxygen in the benzoquinone group forms a hydrogen bond with Y273. The oxygen in the methyl acetate group forms a hydrogen bond with G271. Additionally, its 1H-imidazole forms one π–π interaction with Y264 of PLpro.

The pyrrole group in NSC679525 prefers docking into allosteric site 2 (Figure 9B, lower panel). NSC679525 forms a π–π interaction with R138 of PLpro, while the negatively charged nitrogen in the pyrrole group forms a hydrogen bond with Y71. The oxygen in the (methylsulfonyl)methane group forms a hydrogen bond with N146. Additionally, the oxygen in the cyclohexa-3,5-diene-1,2-dione group forms a hydrogen bond with Y83.

Based on the docking results and kinetic experiments, our data suggest that NSC338106 binds to allosteric sites 1 or 2. Consistently, our kinetic data indicated that NSC338106 inhibits PLpro in an uncompetitive mode, suggesting that the compound binds to a site other than the active site. In contrast, kinetic data indicated that NSC651084 inhibits PLpro through a competitive mode, implying that NSC651084 binds to the active site to compete with substrate binding, which also agrees with our docking result. Finally, our docking study suggests that NSC679525 binds to allosteric site 2, supported by our kinetic data, indicating that NSC679525 inhibits PLpro in a non-competitive mode, implying that it does not bind to the active site.

## 4. Discussion

PLpro of SARS-CoV-2 is an attractive target for antiviral development due to its role in viral polyprotein maturation, assembly of the replication–transcription complex, and interference with the host innate immune response [20,21,53]. We developed a novel HTS assay based on ISG15 as the PLpro substrate and conducted a high-throughput screening of a library containing 1584 synthetic compounds.

Several reports have indicated that ISG15, which contains LXGG motif at its C-terminal end, can be specifically recognized and cleaved by PLpro [26,47,54,55]. Therefore, we hypothesized that ISG15 could serve as a substrate for in vitro assays. We generated a CFP-YFP-based substrate, CFP-3C-ISG15-YFP, in which CFP was linked to YFP through a 3C-ISG15 linker, and used this substrate to assess the proteolytic activity of PLpro via FRET-based analysis. Previously, the CFP-peptide-YFP FRET modality has been used to identify compounds that inhibit protease activity [50,56]. GFP mutants, CFP and YFP, have been engineered for longer wavelengths and are now frequently utilized in FRET applications. CFP, with a Y66W mutation, achieves excitation and emission peaks at 436 and 476 nm, respectively. YFP, designed based on GFP’s crystal structure, incorporates a T203Y mutation which induces a π–π stacking interaction between Thr203 and Tyr66, shifting the spectra with the excitation and emission peaks at 516 and 529 nm, respectively. This mutation also allows the CFP-YFP pair to facilitate FRET detection over greater distances. Based on a similar approach, we designed our substrate and used it to screen for compounds that could inhibit PLpro activity. HTS with this substrate identified twenty-four hits, three of which—NSC338106, 651084, and 679525—showed efficient antiviral activity with EC_50_ values in the micromolar range. We further validated the potency of selected compounds through PAGE-based analysis of the cleavage product of the CFP-3C-ISG15-YFP substrate by PLpro. All three compounds inhibited substrate cleavage. Our results indicate that we have successfully developed a CFP/YFP-based FRET PLpro assay suitable for HTS. Although the compounds we identified have lower potency compared to some PLpro inhibitors reported earlier by others [32], our proof-of-concept study mainly focused on assay development and screened only a small compound library composed of 1584 compounds. Further screening of larger libraries could potentially identify novel and more potent compounds. Additionally, further medicinal optimization of these identified compounds will be necessary to improve their potency and pharmacological properties.

Studies have shown that PLpros of coronaviruses share structural and functional similarities with human DUB enzymes [57], which allows PLpro to cleave not only ubiquitin but also ubiquitin-like modifiers such as ISG15. Since PLpro exhibits DUB enzyme activity, inhibitors of PLpro may also affect human DUBs, making these human enzymes potential off targets. While previous studies showed that identified inhibitors did not inhibit the human DUB activity [57], we assessed the inhibition of a representative human DUB, USP25, with our selected compounds. NSC338106, NSC651084, and NSC679525 inhibited USP25 activity with IC_50_ values above 20 µM. The compounds selected above inhibited PLpro activity with IC_50_ values below 10 µM and exhibited inhibitory activity on SARS-CoV-2 proliferation with EC_50_ values below 5 µM. Therefore, we consider these compounds eligible for further medicinal chemical optimization. Further investigation and optimization are warranted to improve the specificity and efficacy of these compounds as antiviral agents targeting SARS-CoV-2 PLpro while minimizing off-target effects on human DUBs.

Cytotoxicity is a significant concern when dealing with synthetic compound inhibitors, given the homology between PLpro of SARS-CoV-2 and several human cysteine proteases. This similarity can lead to off-target effect and significant cytotoxicity [36]. Consistent with this concern, the three hit inhibitors exhibited moderate cytotoxicity with CC_50_ values of 67.0 µM, 60.3 µM, and 18.6 µM, correspondingly. Importantly, these values were more than 10-fold higher than their respective EC_50_ values. The compound NSC679525 stands out as a potential candidate for further modification as a potent inhibitor for SARS-CoV-2. It is noted that the EC_50_ value for NSC679525 is 0.4 µM, as determined by PRA, whereas it is 2.8 µM by IFA. The discrepancy could arise from the different assays used, with PRA determining live viruses and IFA quantifying the expression of viral proteins. Additionally, we cannot rule out the possibility that NSC679525 may target other pathways, which will require further investigations.

It is a well-known fact that SARS-CoV-2 mutates rapidly, leading to the emergence of newer variants with varying pathogenicity [58,59]. The mutations in the gene encoding PLpro always pose a challenge, especially when they occur in the binding site of inhibitors. A study conducted by Perlinska et al. revealed that nearly 5% of the 70,000 analyzed sequences of PLpro, spanning nsp3 residues from 746 to 1060, exhibited mutations resulting in amino acid changes [60]. Further analysis with the known inhibitor, GRL0617, demonstrated that mutations within the PLpro domain such as P992S, E1008D-Y1009H, and T1010A-Y1013C could potentially influence both inhibitor binding and the overall activity of the protein. Additionally, several other mutations in the nsp3 gene that encodes PLpro have been identified in emerging strains of the virus, such as K38R, L1266I, and A1892T in the Omicron variant [59]. Therefore, we performed cell-based antiviral assays to assess the efficacy of the identified PLpro inhibitors against two different viral variants, namely, the wild-type WA1 strain and the Omicron (B.1.1.529) variant. These two variants differ in terms of their infectivity, transmissibility, and ability to evade the host immune response [61]. Our results revealed that the PLpro inhibitors NSC338106, 651084, and 679525, identified by our group, effectively inhibited the replication of both the WA1 and Omicron strains in a dose-dependent manner, with EC_50_ values in the lower micromolar range. The EC_50_ values of these compounds for the Omicron strain are only slightly lower than those for the WA1 WT strain. This result suggests that the three nsp3 mutations (K38R, L1266I, and A1892T) in the Omicron strain, which are located outside the PLpro domain, do not directly affect the compounds’ inhibitory activity. However, we cannot rule out the possibility that these mutations may indirectly influence compound binding, leading to slightly lower antiviral activity against the Omicron strain compared to the WA1 strain. Overall, considering these promising findings, in vivo studies are highly anticipated for the development of an effective PLpro-based antiviral.

To gain a deeper understanding of the mode of action of these protease inhibitors, it would be beneficial to examine the complex formation between PLpro and selected compounds. Although docking-based virtual screening often generates a high percentage of false positives [62], molecular docking is a very useful tool to understand the interaction between the identified compounds and their targets, as well as to help design inhibitors with improved potency [63]. In this study, we used docking to further confirm the mechanism of inhibition. The docking results are consistent with that determined by enzyme inhibition kinetic studies.

In summary, the CFP-3C-ISG15-YFP fusion protein is a unique substrate for identifying potential SARS-CoV-2 PLpro inhibitors using FRET assay. Three inhibitors identified in our study demonstrated efficacy comparable to the previously identified positive control inhibitor, GRL0617, and inhibit PLpro with IC_50_ in the micromolar range. Additionally, compound NSC679525 exhibits inhibition against Omicron and WA1, suggesting its potential for further development. Further studies, including co-crystallization of PLpro with the identified inhibitors and evaluation in animal models, are required to confirm the potency of the inhibitors.

## Figures and Tables

**Table 1:** Characteristics of candidate compounds.

Compounds	IC_50-PLpro_ (µM)	IC_50-PLpro-tw_ (µM)	IC_50-_ _USP25_ (µM)	SI	EC_50-IFA_ (µM)	EC_50-PRA_ (µM)	EC_50-Omi_ (µM)	CC_50_ (µM)	*K_D_* (µM)
Tween-20	−	+	−		−	−		−	+
GRL0617	1.7	2.8							
207895	0.9	5.0							
338106	3.3	3.9	37	11.2	2.9	3.3	6.9	67.0	6.6 (±0.1)
341956	12.4	>200							
651084	4.9	2.9	23	4.7	2.8	4.6	5.9	60.3	13.9 (±0.9)
679525	6.0	11	41	6.8	2.8	0.4	5.4	18.6	4.1 (±2.9)

IC_50_: inhibitor concentration at which a compound inhibits the response up to 50%; PLpro: Papain-like protease, SI: selectivity Index, USP25: ubiquitin-specific proteases 25; EC_50_: effective concentration at which a compound inhibits viral replication by 50%; IFA: immunofluorescence assay; PRA: plaque reduction assay; CC_50_: cytotoxicity concentration of inhibitors required to reduce cell viability by 50%; Omi: Omicron strain; *K_D_*: equilibrium dissociation constant or binding affinity.

**Table 2:** The glide docking scores for each compound docked within the three putative binding sites.

Compounds	Docking Scores			Putative Mode of Action
	Active Site	Allosteric Site 1	Allosteric Site 2	
NSC338106	−6.16	−7.53	−7.25	Uncompetitive
NSC651084	−8.44	−6.8	−7.10	Competitive
NSC679525	−3.79	−6.62	−7.16	Non-competitive

## Data Availability

Data will be available upon request.

## Supplementary Materials

The following supporting information can be downloaded at https://www.mdpi.com/article/10.3390/v16081239/s1. Figure S1: Dose–response curve of GRL0617 tested against PLpro using CIY and peptide-AMC substrate. Figure S2: Primary high-throughput screening (HTS). Figure S3: Dose–response curve of tested compounds with 5 min and 60 min. Figure S4: Dose–response curve of PLpro inhibitors tested against human deubiquitinase, USP25, using a CFP-YFP based substrate. Figure S5: Putative druggable binding sites. Figure S6: Grid boxes used for induced fit. Figure S7: Binding model of NSC338106 to the SARS-COV-2 PLpro in the putative allosteric site 2. Table S1: Time of addition studies. Table S2: Putative druggable binding sites characterization.
