# Supplementary material for: An ISG15-Based High-Throughput Screening Assay for Identification and Characterization of SARS-CoV-2 Inhibitors Targeting Papain-like Protease
Source: Viruses. 2024 Aug 1;16(8):1239. doi: 10.3390/v16081239 (PMC11359932; doi:10.3390/v16081239)
Supplement: Supplementary file 1 [file viruses-16-01239-s001.zip › viruses-3092565-supplementary.pdf]

An ISG15-Based High-Throughput Screening Assay for Identification and Characterization of SARS-CoV-2 Inhibitors Targeting Papain-Like Protease

by Subodh Kumar Samrat [1,*], Prashant Kumar [1], Yuchen Liu [1], Ke Chen [1], Hyun Lee [2], Zhong Li [1], Yin Chen [1] and Hongmin Li [1,3,4,*]

[1] Department of Pharmacology and Toxicology, R Ken Coit College of Pharmacy, The University of Arizona, 1703 E Mabel St, Tucson, AZ 85721, USA; du.pkumar@gmail.com (P.K.); yliu@pharmacy.arizona.edu (Y.L.); kechen2@arizona.edu (K.C.); zli@pharmacy.arizona.edu (Z.L.); ychen@pharmacy.arizona.edu (Y.C.)

[2] Department of Pharmaceutical Sciences, College of Pharmacy and Biophysics Core, Research Resources Center, University of Illinois at Chicago, Chicago, IL 60607, USA; danielhl@uic.edu

[3] Department of Chemistry and Biochemistry, College of Science & College of Medicine, The University of Arizona, Tucson, AZ 85721, USA

[4] The BIO5 Institute, The University of Arizona, Tucson, AZ 85721, USA

* Correspondence: subodhsamrat@arizona.edu (S.K.S.); hli001@arizona.edu (H.L.);
Tel.: +1-520-621-5789 (S.K.S); +1-520-621-5728 (H.L.)

Supplementary Figure S1.

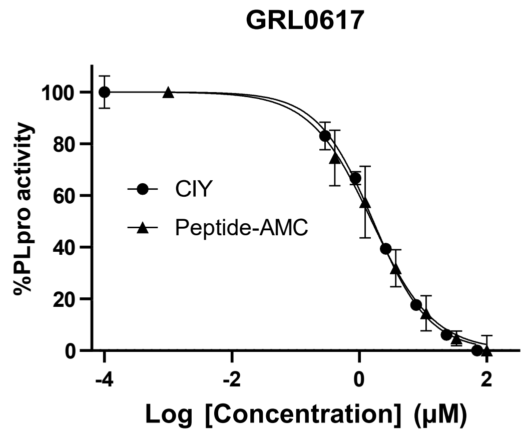

Supplementary Figure S1: Dose response curve of GRL0617 tested against PLpro using CIY and peptide-AMC substrate. CFP-ISG15-YFP and peptide-AMC substrates were used to calculate IC_{50} of GRL0617 compound against PLpro. We obtained IC_{50} of 1.6 μM with the CIY substrate and 1.5 μM with the peptide-AMC based substrate.

Supplementary Figure S2.

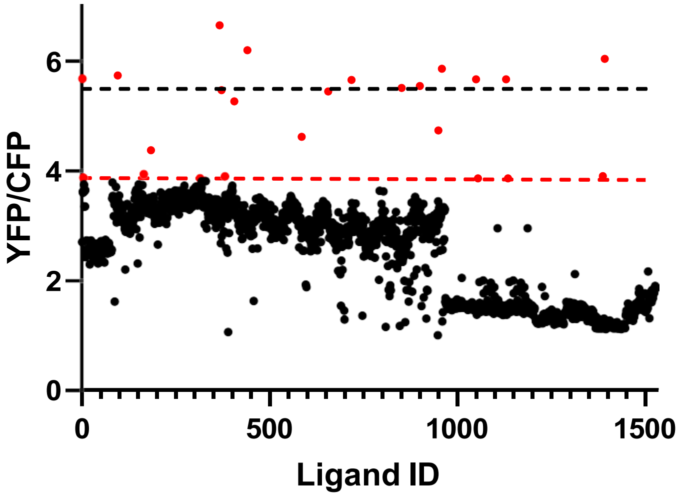

Supplementary Figure S2: Primary high-throughput screening (HTS). YFP/CFP ratio scatter plot. The red line indicated threshold of our experiment (70% inhibition) and black line indicated average of positive control.

Supplementary figure S3.

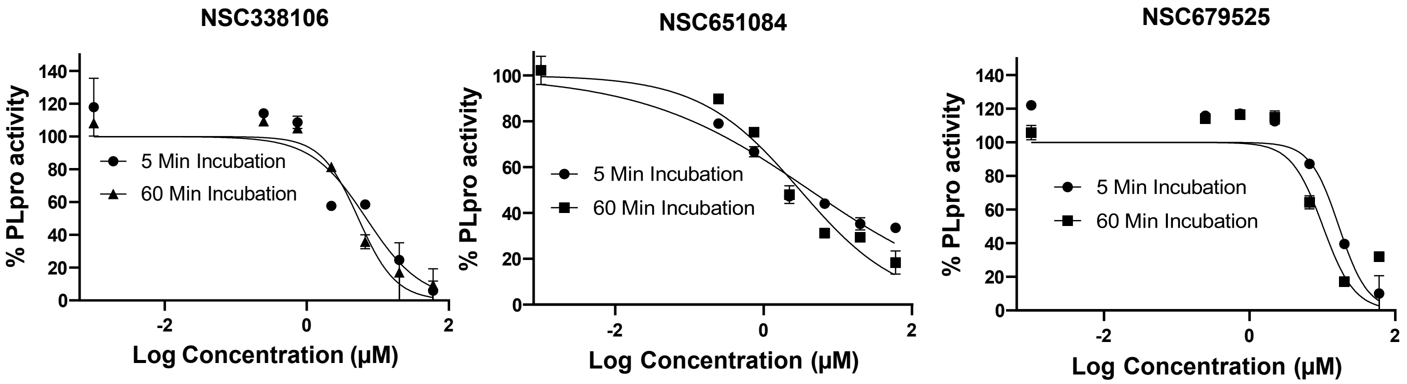

Supplementary Figure S3: Dose response curve of tested compounds with 5 min and 60 min incubation. CIY substrate was used to calculate IC_{50} of compounds. IC_{50} are mentioned in the Supplementary Table 1.

Supplementary Table S1

	IC_{50} (µM)	
	5 Min Incubation	60 Min Incubation
Compounds		
NSC 338106	6.9	5.3
NSC 651084	4.2	3.1
NSC 679525	16.8	10.2

Supplementary Figure S4.

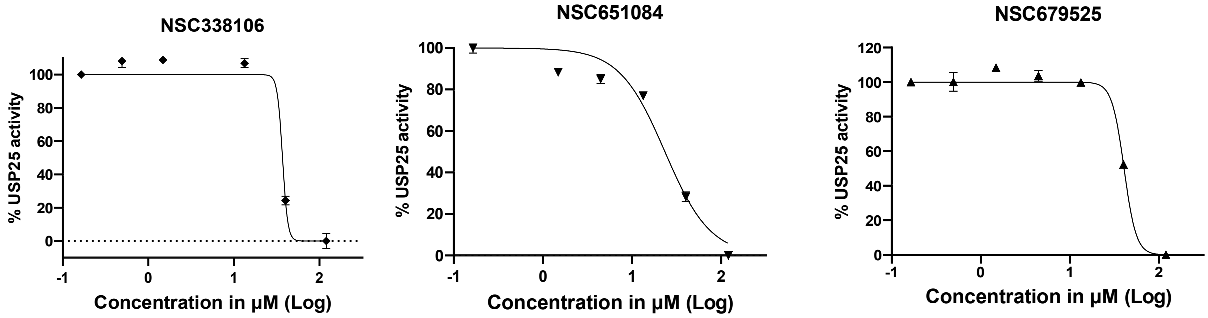

Supplementary Figure S4: Dose response curve of PLpro inhibitors tested against human deubiquitinase, USP25, using a CFP-YFP based substrate. This assay is performed exactly as outlined in the protocol for the PLpro enzyme. IC_{50} of each inhibitor was determined. (**A**) NSC338106, IC_{50}, 37 µM, (**B**) NSC651084, IC_{50}, 23 µM and (**C**) NSC679525, IC_{50}, 41 µM. All experiments were performed in triplicate and all data are expressed as the mean ± standard deviation.

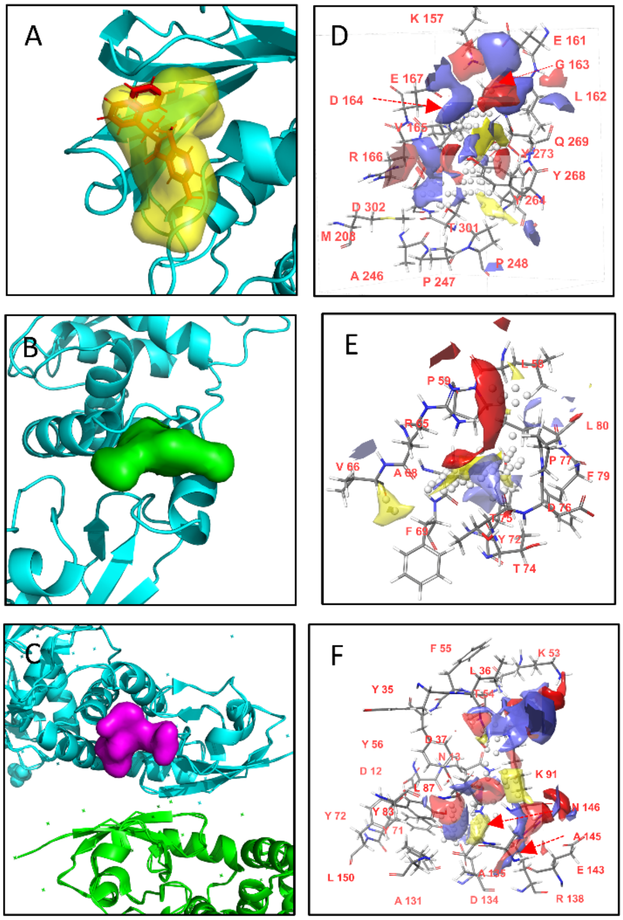

Supplementary Figure S5: Putative druggable binding sites. Three druggable binding sites were identified and characterized using Maestro Sitemap based on Crystal structure of the wild type of SARS-CoV-2 papain-like protease (PLPro) with inhibitor GRL0617(PDB ID: 7JRN). The active site is colored yellow. Allosteric site 1 is colored in green and allosteric site 2 is colored in magentas. (**A**) Active site. GRL0617 is marked in red which locates in our best putative binding site. (**B**) Allosteric site 1. (**C**) Allosteric site 2. Allosteric site 2 locates near dimerization interface. (**D-F**) Surface characterization for each binding site. The H-bonding acceptor surface is marked in red, the H-bonding donor surface is marked in blue, and the hydrophobic surface is marked in yellow. White balls indicate the pocket space. The residues contributing to the surface are indicated in stick representation.

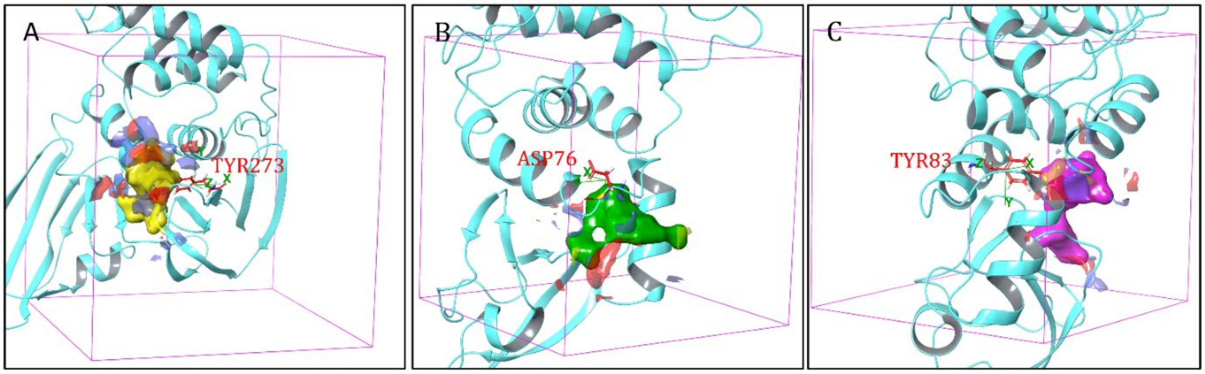

Supplementary Figure S6. Grid boxes used for induced-fit docking. Three grid boxes are created around the centroid of PLpro putative ligand binding pockets for induced-fit docking. (**A**) Active site grid box with the centroid of TYR273 within a 30 Å radius. The active site is marked in yellow, and TYR273 is represented as a red stick. (**B**) Allosteric site 1 grid box with the centroid of ASP76 within a 20 Å radius. The putative allosteric site 1 is marked in green, and ASP76 is represented as a red stick. (**C**) Allosteric site 2 grid box with the centroid of TYR83 within a 20 Å radius. The putative allosteric site 2 is marked in magenta, and TYR83 is represented as a red stick.

Supplementary Table S2. Putative druggable ligand binding sites characterization. SScore indicates the site score. Dscore indicates the druggability score. Residues indicates the residues contributing to the putative ligand binding sites.

Putative site	Sscore	Dscore	Residues
Active site	0.937	0.931	K157, E161, L162, G163, D164, V165, R166, E167, M208, A246, P247, P248, Y264, Y268, Q269, Y273, T301, D302
Allosteric site 1	0.883	0.918	L58, P59, R 65, V66, A68, F69, Y72, T74, T75, D76, P77, F79, L80
Allosteric site 2	0.881	0.872	D12, N13, Y35, L36, D37, K53, T54, F55, Y56, Y71, Y72, Y83, L87, K91, A131, D134, A135, R138, E143, A145, N146, L150

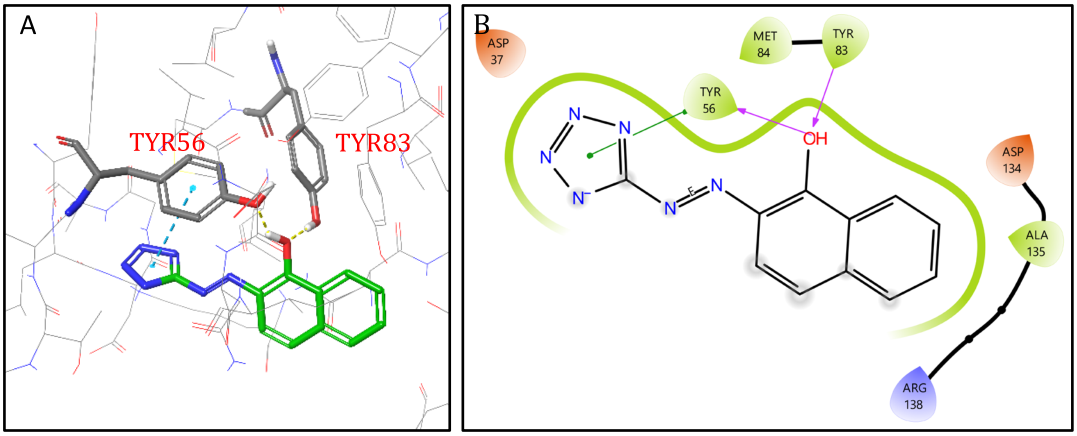

Supplementary Figure S7. Binding model of NSC338106 to the SARS-COV-2 PLpro in the putative allosteric site 2. NSC338106 is docked into allosteric site 2. (**A**) 3D interaction maps, with dashed lines indicating different interaction types by color: yellow for hydrogen bonding and cyan for π-π interactions. (**B**) 2D-Interaction maps. π-π interactions are indicated in green and hydrogen bonding is indicated in purple with arrow(s) indicating role as donor or acceptor.
